# Inter-professional delirium education and care: a qualitative feasibility study of implementing a delirium Smartphone application

**DOI:** 10.1186/s12911-016-0288-1

**Published:** 2016-04-30

**Authors:** Melvyn Zhang, Kathleen Bingham, Karin Kantarovich, Jennifer Laidlaw, David Urbach, Sanjeev Sockalingam, Roger Ho

**Affiliations:** Biomedical Global Institute of Healthcare Research and Technology (BIGHEART), National University of Singapore, Singapore, Singapore; Department of Psychiatry, University of Toronto, Toronto, Ontario Canada; Department of Psychological Medicine, Yong Loo Lin School of Medicine, National University of Singapore, Singapore, Singapore; Institute of Mental Health, 10 Buangkok Green Medical Park, Singapore, Singapore

**Keywords:** Delirium, Education, E-health, M-health, Smartphone applications

## Abstract

**Background:**

Delirium is a common medical condition with a high prevalence in hospital settings. Effective delirium management requires a multi-component intervention, including the use of Interprofessional teams and evidence-based interventions at the point of care. One vehicle for increasing access of delirium practice tools at the point of care is E-health. There has been a paucity of studies describing the implementation of delirium related clinical application. The purpose of this current study is to acquire users’ perceptions of the utility, feasibility and effectiveness of a smartphone application for delirium care in a general surgery unit. In addition, the authors aimed to elucidate the potential challenges with implementing this application.

**Methods:**

This quantitative study was conducted between January 2015 and June 2015 at the University Health Network, Toronto General Hospital site. Participants met inclusion criteria if they were clinical staff on the General Surgery Unit at the Toronto General Hospital site and had experience caring for patients with delirium. At the conclusion of the 4 weeks after the implementation of the intervention, participants were invited by email to participate in a focus group to discuss their perspectives related to using the delirium application

**Results:**

Our findings identified several themes related to the implementation and use of this smartphone application in an acute care clinical setting. These themes will provide clinicians preparing to use a smartphone application to support delirium care with an implementation framework.

**Conclusions:**

This study is one of the first to demonstrate the potential utility of a smartphone application for delirium inter-professional education. While this technology does appeal to healthcare professionals, it is important to note potential implementation challenges. Our findings provide insights into these potential barriers and can be used to assist healthcare professionals considering the development and use of an inter-professional clinical care application in their setting.

## Background

Delirium is a common medical condition with a high prevalence in hospital settings, with rates ranging between 6 and 56 % depending on the patient population [1]. If not recognized early, delirium leads to increased morbidity and mortality [2]. Despite its prevalence and serious sequelae, it often goes unrecognized. Moreover, effective delirium management requires a multi-component intervention, including the use of interprofessional teams and evidence-based interventions at the point of care [3].

Given the need for improved delirium recognition and collaborative management, there has been an increasing focus on delirium education interventions. However, inter-professional delirium training is typically offered as a one-time educational intervention [4]. Whilst these conventional programs might be effective in providing clinicians and healthcare professionals with increased knowledge and skills pertaining to the recognition and the treatment of delirium, prior studies have highlighted the inherent limitations of such one-time intervention [4]. Recent studies and reviews have recommended the development of point of care practice tools that clinicians across professions could use in addition to other delirium education components [4]. It is believed that with such tools, clinicians will be more competent in managing delirium, resulting in improved health outcomes.

One vehicle for increasing access of delirium practice tools at the point of care is E-health [5]. The World Health organization defines E-health as the process in which health resources and healthcare are being communicated and transferred by electronic media [6]. Over the last decade, there has been a massive advance in E-health, which has led to the creation of multiple online learning platforms for clinicians and allied healthcare professionals to stay abreast of the latest treatment algorithms and guidelines. Since 2010, there has been an accompanying advancement in M-health as well, which refers to the usage of mobile and smartphone technologies in healthcare. With the growth and advancement in M-health, smartphone are being increasingly used medical disciplines [6], surgical disciplines [7] and psychiatry [8]. It is thus not surprising that with the introduction of smartphones, healthcare professionals are beginning to turn to smartphone applications to aid them in clinical assessment and management [6]. Prior studies have highlighted the utility of smartphone applications as quick and interactive platforms that are time saving and allow for increased confidence and accuracy, as well as error reduction in the detection and management of various clinical conditions [6]. By providing instant access to medical information, it is postulated that applications not only improve communication amongst staff, but help healthcare professionals in decision-making. The cumulative effect of these factors would be the reduction in the overall economic costs incurred by the healthcare organization [6].

A review of the current published literature using the keywords “Delirium, smartphone” revealed that there are only three studies published to date about the perception of the usefulness of a smartphone application. Zhang et al. [9] described the development of a delirium educational resource application that contains guideline-based information on the assessment and management of delirium. This delirium application also includes practice tools developed to assist clinicians at a Canadian healthcare institution. Initial results, based on a sample of 19 clinicians and allied healthcare professional demonstrated that there was a shift in the confidence level of the participants after they used the smartphone applications [9]. Zhang et al. [9] also managed to acquire healthcare user perceptions about the application in their study. Tieges Z et al. (2015) [10] described the potential of a smartphone application in the objective detection of attentional issues in delirium. Sangha S et al. (2015) [11] highlighted how a smartphone application could potentially be helpful in terms of cognitive assessment. Whilst there are to-date only these published studies, there are a multitude of delirium related applications on the respective application stores. It would be timely if there were a scoping review or a content analysis looking into the individual features of these applications and evaluating their information quality.

There are limitations that could potentially impact the receptiveness of smartphone technology in healthcare. Baig et al. (2015) [12] reviewed a series of mobile healthcare applications (aside to delirium applications) and identified several limitations that could impact the receptiveness of smartphone applications in medicine. These challenges include issues related to reliability, efficiency and acceptability. Their review identified the need for smartphone applications to be user-centered in design in order for them to be more widely used and accepted. In addition, the availability of the same application on different platforms could potentially increase its use in practice. To date, most of the smartphone applications are programmed specifically for an individual platform. Other factors such as security and privacy also limit the use of smartphone applications in medical settings.

The purpose of this current study was to evaluate users’ perceptions of the utility, feasibility and effectiveness of a smartphone application for delirium care in a general surgery unit. The general surgery unit was selected due to its high prevalence of delirium and the unit’s prior exposure to an inter-professional delirium training program [13]. In particular, we aimed to elucidate the potential challenges with implementing this application. Our study used qualitative methods to explore the following research questions:

- What are the perspectives of healthcare professionals regarding the implementation and availability of a delirium educational smartphone application?
- What are some of the key challenges and limitations with regard to the implementation and utilization of such a smartphone application for clinical care?

## Methods

### Study design

This qualitative study was conducted between January 2015 and June 2015 at the University Health Network, Toronto General Hospital site. In preparation, the authors ran a search using the keywords “delirium” and “education” in both the Apple and Android application stores. Although several delirium applications on both stores have education components, they are limited to general information with little information to assist clinicians in caring for delirium patients.

Participants met inclusion criteria if they were clinical staff at the Toronto General Hospital site and had experience caring for patients with delirium. Participants in the study could be nurses and other healthcare professionals (for example, physicians, physiotherapists, occupational therapists or social workers). There were no exclusion criteria.

The delirium application used in this study has been previously described in the literature [11]. The design and conceptualization of the delirium clinical care application was based on the feedback from an inter-professional delirium committee that comprises of both clinicians and allied healthcare professionals, from the respective specialties. The inter-professional committee contributed their expertise with regards to the contents of the educational application. The smartphone application that was conceptualized contained not only links to evidence based delirium care guidelines, but also assessment toolkits commonly used for delirium care. In addition, the smartphone application also contains flash-cards of commonly used medications as well as non-pharmacological treatment guides. In addition, the application also contains a patient education information guide in order for healthcare professionals to provide bedside education with patients and families.

The delirium application was launched after clinical staff was oriented to its features and use. Staff was asked to use the delirium smartphone application for the next 4 weeks as part of their clinical care of delirious patients. Clinicians continued to receive weekly notices over the 4 week from the unit manager highlighting the delirium application’s role as a delirium care resource.

At the conclusion of the 4 weeks, participants were invited by email to participate in a focus group to discuss their perspectives related to using the delirium application. Focus groups used a semi-structured interview guide consisting of five questions regarding the barriers and facilitators of the delirium application, suggested changes to the application, participants’ general perception of medical applications and their implementation at work, and examination of specific cases in which the application was used (see Appendix). Two focus groups were held with 3 and 7 participants in each group and each focus group lasted approximately 30 min.

### Data analysis

Focus groups were facilitated by two individuals: a research assistant and a psychiatry resident. They were audio recorded and transcribed verbatim. Focus group transcripts were analyzed using a grounded theory informed approach [14, 15], consisting of open, axial and selective coding to generate higher order themes. Two researchers reviewed the transcripts and identified themes and sub-themes and a third researcher facilitated the consensus process. After the thematic framework was generated, the focus group interviews were analyzed using HyperResearch™.

## Results

### Focus group data

Ten nurses from the General Surgery unit, who were separate from the 19 questionnaire participants, volunteered to participate in two focus groups to identify the potential barriers and facilitators to delirium application usage in clinical practice. Focus group thematic analysis is summarized in Table 1.

**Table 1:** Thematic Analysis of the utility and challlenges with regards to the implementation of a Delirium Clinical Application

Thematic analysis	Qualitative feedback
Embedding Smartphone Applications into Existing Technological Infrastructure	
a. Device Compatibility	“I found it difficult to use on my phone. My phone is outdated”
b. Integration into Existing Device Types	“Some people might be more inclined to go into it if it was already embedded into [the electronic patient record]. It was just a click away, versus trying to bring it up on a completely different device.”
c. Wifi Accessibility and Data Usage	“Not at lot of people have data, or maybe most of them, but some of them don’t. And then if you use Wi-Fi, some of the connection is not right.”
Incorporating Smartphone Use into Unit Culture	
a. Infection Control	"I don’t ever take my phone out near the bedside….I mean I don’t really wanna touch my phone when I’m around the bedside"
b. Professionalism	“It would seem a bit strange to my patients if I was on my phone” "But the only issue is that I don’t want to pull the phone out, its not a part of the culture."
c. Embedding Smartphone Use into Practice Culture	"…we don’t really use our phones in practice. It’s not like us to be whipping out your personal phone at the bedside …. So, I don’t know that it’s handier for us to have it on a phone versus…as part of like… within the [hospital internet] under say 'clinical tools”.
Supporting Education and Practice	
a. Accessibility of Information Retrieval	‘We can access iPhones faster than these computers”
b. Focusing on Patient/Family Education	“There could be something very helpful. Um… like I don’t know… like teaching the family members on how to do different things."
c. Evidence Based Practice	"…it needs to provide something additional and evidence based and simple and easy to use, like not extraneous information."
Design of Smartphone Application	
a. Navigability	“There were a lot of categories. It was hard to navigate at first.”
b. Strategic Use of Text	“It was a bit wordy, I think there was a lot of text.”
Interprofessional Components	
a. An Inclusive Healthcare App	“I think when people hear medical they associate that term with physician and so if it is a healthcare application, it’s inclusive of everybody.”
b. Content Relevant to All Professions	“Then you just have to make sure that the content kind of translates to all fields as well, and it’s not just something that physicians would find useful.”

Five main themes emerged from the focus group transcript analysis: i) embedding smartphone applications into existing technological infrastructure, ii) incorporating smartphone use into workplace culture, iii) the potential for smartphone applications to support education and practice, iv) inter-professional components of application development and v) design of smartphone application. Themes and sub-themes with examples are summarized in Table 1.

Participants identified the need to embed the smartphone application into existing technological infrastructure in order to optimize its use. Nurses identified challenges with device compatibility, specifically the presence of outdated phones and difficulty accessing the application reliably. An additional sub-theme emerged in this area: the potential integration of the application into existing electronic health records, given that nurses were already accessing this platform as part of their clinical work. In addition, implementation and use of the delirium application was also affected by concerns about data usage and access to free wifi connections within the hospital unit.

A second theme emerging from the focus groups were concerns about incorporating the delirium smartphone application into the existing general surgery unit culture. For example, participants expressed concerns about infection control with using their own phones at the bedside. Respondents suggested that using other devices dedicated to the unit for clinical use might address this issue. Moreover, nurses indicated that they had concerns about professionalism with the application, as they worried about patients perceiving nurses negatively for using their personal phones at the bedside. Participants commented that clinical and educational tools were historically available on the intranet on hospital computers, and this means of accessing electronic data was more in keeping with the unit’s culture.

Despite the challenges associated with implementing the application on the unit, participants noted that there are some potential benefits to accessing information via smartphone. A third theme was the role of the smartphone application to support education and clinical practice. Participants noted that the application provided faster access to information and delirium resources. They also noted benefit in having patient and family education resources on the phone. Nurses described the benefit of having evidence-based content in one location, but, in a fourth theme, suggested that content that should be more accessible with less text and easier to navigate for specific information. One of the difficulties the authors faced in the conceptualization of the smartphone application was designing it to be compliant across a variety of smartphone operating systems. Given that a high percentage of healthcare professionals in Canada still use Symbian and Blackberry based phones, it was challenging to build an application that functions well across different systems. Hence, the authors resorted to the creation of a responsive web-based smartphone application. This led to a lack of flexibility in customization of the interface and may have resulted in the navigation difficulties noted by the focus group.

The final theme was the need for the smartphone application to be constructed with an inter-professional audience in mind. Participants felt that the application needed to be inclusive of all healthcare professionals and content should be tailored to meet the needs of a range of healthcare professions.

## Discussion

This is one of the first studies to describe perceptions and user experiences with an inter-professionally developed smartphone application to support delirium management in an acute care setting. Our findings identified several themes related to the implementation and use of this smartphone application in an acute care clinical setting. These themes will provide clinicians preparing to use a smartphone application to support delirium care with an implementation framework.

With the massive advances in technologies, there has been increased adoption of technology for inter-professional medical education. With regard to delirium education, Chao et al. (2012) [16] have previously evaluated the potential of an online curriculum in teaching delirium management to medical students. The students were receptive to the online curriculum and this innovative teaching method was found to be equally effective to a conventional lecture. This study highlights the feasibility of having an online platform for education in delirium education and management. A more recent study by Middle and Miklancie (2015) [17], suggested that in order to improve nursing professionals’ knowledge regarding delirium recognition, educational strategies should focus more on teaching health professionals about diagnostic tools, as well as implementing a structured delirium educational program. We suggest that our findings corroborate previous research demonstrating health care professionals’ positive response to innovative educational strategies. We propose that this is due to the ability for the application to provide structured and evidence-based material in a highly accessible manner.

We have identified several challenges with its implementation. Of note, Baig et al. (2015) [12] highlighted similar challenges. They noted issues with reliability of the application and generalizability across various smartphone platforms, and highlighted the importance of having end users involved in the initial application conceptualization process to ensure that the design is user-centred.

The challenges that we elicited from our focus group data are valuable since they inform future research and application development. Focus group participants identified the need to embed smartphone applications into existing technological infrastructure, for example, through integration with electronic health records. Furthermore, participants highlighted several challenges in incorporating smartphone devices into their unit, specifically with regard to infection control and professionalism. They also highlighted and surfaced concerns regarding the breadth and depth of the information included, specifically having too much text information to apply in a meaningful way at the patient’s bedside. Despite the challenges discussed above, focus group participants highlighted the advantages of smartphone applications, including the potential for rapid access to evidence-based material regarding delirium treatment and management, both for clinical practice and patient education at the point of care.

The main strength this study is that it supports clinician acceptance and feasibility of using novel technologies for inter-professional delirium care. We have demonstrated that a smartphone application could be an intervention tool that improves helps healthcare professionals in delirium management. These results are in keeping with previous studies demonstrating the feasibility of an online educational portal for delirium education [13].

Nevertheless, there are inherent limitations in our current study. Our sample size was small, meaning that our findings should be viewed as preliminary. Furthermore, our data comes from healthcare professionals who owned a smartphone and could use it in their day-to-day clinical work. Our focus group participants were nurses who worked on the general surgery unit. Thus, additional studies are needed to determine whether our findings would generalize to other healthcare settings and specialties. Moreover, we have not managed to capture the absolute amount of time that our participants have spent using the application. We have also not compared the amount of time they have send using our application against other healthcare applications. The authors acknowledge that the questions we have asked might not be comprehensive enough to capture concerns pertaining to the integration of M-health in delirium education at the bedside. The results which the authors have acquired have not assessed how such an application could have improved clinical care, as there is no further analysis done to determine if this results in a reduction in the absolute length of stay. Lastly, the authors have not made use of validated questionnaires to determine user perception quantitatively.

## Conclusions

While most of the current studies published have supported the efficacy of mobile applications in clinical care, our current findings demonstrate that while this modality of technology does appeal to healthcare professionals, it is also key to note potential implementation challenges. The integration of mobile applications in clinical settings needs to take into account the needs of the end user. Our findings provide insights into these potential barriers and could be used to assist healthcare professionals considering the development and use of an inter-professional clinical care application in their setting.

## Ethics approval and consent to participate

This study received approval by the University Health Network Research Ethic Board. All participants were explained the study details and given time to review the consent form before providing written informed consent.

## Consent for publication

Not applicable.

## Availability of data and materials

The data-set which we have acquired will not be shared as a supplementary file. Our ethical approval does not permit the sharing of the entire dataset which we have acquired.

## Appendix

### Focus group questions

1. What did you find to be the most useful feature of the app? What did you like the best about the app?
2. What was least useful feature of the app? Why?
3. What additions or changes would you make to the app to improve it?
4. What is your opinion on medical apps, specifically using them while at work? Probe: What was it like using this app to care for your delirious patients?
5. Describe a situation in which you used this app. What role did the app play in helping you handle the situation? Do you think using the app made a difference?

## Abbreviations

E-Health: electronic health
M-Health: mobile health
